# An Innovative Approach to Developing Educational Materials for Veterans’ Caregivers using Co-Design

**DOI:** 10.1093/geroni/igaf122.2973

**Published:** 2025-12-31

**Authors:** Jacqueline Boudreau, Lynette Kelley, Aileen McGrory, Marianne Desir, Eileen Dryden

**Affiliations:** Veterans Health Administration, Bedford, Massachusetts, United States; Veterans Health Administration, Aurora, Colorado, United States; Veterans Health Administration, Bedford, Massachusetts, United States; Veterans Health Administration, Miami, Florida, United States; Veterans Health Administration, Bedford, Massachusetts, United States

## Abstract

Informal caregivers are invaluable members of many older adults’ care teams. Supporting caregivers improves their wellbeing and engagement with patient care teams, improving patient outcomes including longevity of aging in place. The Veterans Health Administration (VA) houses an array of caregiver resources which many caregivers are unaware of. Further, caregiver perspectives have had limited integration into caregiving educational materials. Thus, to increase caregiver engagement and connection to resources, we co-designed caregiver-facing educational materials with caregivers of VA-enrolled Veterans. Caregivers were oriented to project goals and design principles. They contributed to every dimension of development: content, messaging, design, and tailoring by media type. Researchers and caregivers used these dimensions to structure monthly virtual meetings, during and after which researchers summarized and incorporated caregiver feedback, noting any exclusions and rationale (e.g., outside of project’s scope). Caregivers noted that meetings were well organized and enjoyed paying their knowledge forward to help peers. Reviewing how their feedback was integrated made caregivers feel valued. Orientation to design and live edits helped caregivers envision their suggestions. Given that caregivers value learning from peers, materials made with caregivers for caregivers are likely to be more appealing, accessible, and useful. Notably, our four caregiver co-designers were White; three were wives and one an adult daughter of VA-enrolled Veterans. Future research should replicate this process with caregivers in different contexts. Co-design with recent caregivers provides them a meaningful experience and leverages their collective wisdom to empower other caregivers to sustain themselves and the older adults they care for.

